# Galectin-3 and Coronary Artery Disease: An Inflammation-Based Approach

**DOI:** 10.3390/jcm15103712

**Published:** 2026-05-12

**Authors:** Rıdvan Bora, Rojda Tanrıverdi, Şenay Balcı Fidancı, Burak Toprak, Cemil Gülüm, Oben Döven, Lülüfer Tamer

**Affiliations:** 1 Department of Cardiology, Mersin City Education and Research Hospital, 33800 Mersin, Turkey; dr.ridvanbora@outlook.com; 2 Department of Biochemistry, Mersin University Faculty of Medicine Hospital, 33110 Mersin, Turkey; rjd_tnv_73@hotmail.com (R.T.); senaybalci1980@hotmail.com (Ş.B.F.); cemilgulum@gmail.com (C.G.); lutamer@yahoo.com (L.T.); 3 Department of Cardiovascular Surgery, Mersin City Education and Research Hospital, 33800 Mersin, Turkey; 4 Department of Cardiology, Mersin University Faculty of Medicine Hospital, 33110 Mersin, Turkey; obendoven@icloud.com

**Keywords:** Galectin-3, atherosclerosis, inflammation, coronary artery disease

## Abstract

**Background/Objectives:** Coronary artery disease is a chronic inflammatory disorder characterized by progressive atherosclerosis and heterogeneous clinical presentations ranging from acute coronary events to stable ischemic conditions. Galectin-3 is a β-galactoside-binding lectin involved in inflammatory responses, fibrosis, and tissue remodeling, and has been investigated as a potential biomarker in cardiovascular diseases. However, its diagnostic significance across different clinical stages of coronary artery disease remains unclear. **Methods:** This prospective study included 180 participants who underwent coronary angiography and were classified into three groups: control (n = 60), acute coronary syndrome (n = 60), and chronic coronary syndrome (n = 60). Serum Galectin-3 concentrations were measured using an enzyme-linked immunosorbent assay. Group comparisons were performed using non-parametric statistical tests. Correlation analysis, receiver operating characteristic curve analysis, and multivariable logistic regression were conducted to evaluate diagnostic performance and independent associations. **Results:** Galectin-3 concentrations were significantly higher in both acute coronary syndrome and chronic coronary syndrome groups compared with the control group (*p* < 0.001), whereas no significant difference was observed between the two disease groups. Receiver operating characteristic analysis demonstrated limited diagnostic performance for identifying acute coronary syndrome (area under the curve 0.617, sensitivity 96.7%, specificity 43.3%, *p* = 0.027) and poor diagnostic performance for chronic coronary syndrome (area under the curve 0.541, sensitivity 91.7%, specificity 30.0%, *p* = 0.436). In multivariable analysis, Galectin-3 was not identified as an independent predictor of either clinical condition. Age and smoking were independently associated with acute coronary syndrome, while age and male sex were independently associated with chronic coronary syndrome. **Conclusions:** Galectin-3 levels are elevated in patients with coronary artery disease and appear to reflect the inflammatory burden associated with atherosclerosis. However, its diagnostic discrimination between different clinical stages of coronary artery disease remains limited. Larger prospective studies are required to clarify its clinical value.

## 1. Introduction

Coronary artery disease (CAD) is a clinical condition characterized by myocardial ischemia resulting from the accumulation of atherosclerotic plaques in the epicardial coronary arteries [1]. At the core of its pathophysiology lies atherosclerosis, a chronic disorder driven by the deposition of lipids, infiltration of inflammatory cells, and multiple biological mediators within the vascular wall [2]. These processes impair vascular integrity, cause narrowing of the arterial lumen, and ultimately disrupt the balance between myocardial oxygen supply and demand. Clinically, this pathophysiological mechanism may present as chronic coronary syndrome (CCS) or as acute coronary syndrome (ACS) [3]. ACS and CCS represent different stages of the atherosclerotic spectrum. In ACS, acute plaque rupture and thrombus formation are predominant, whereas CCS is usually associated with stable plaque morphology and persistent low-grade inflammation [3]. These distinctions may also be reflected in biomarker levels.

Galectin-3 (Gal-3) is a β-galactoside-binding lectin that plays essential roles in processes such as cell–cell and cell–matrix interactions, proliferation, differentiation, and regulation of inflammatory responses [4]. Galectin-3 is clinically available as a biomarker and has been widely investigated for prognostic stratification in cardiovascular conditions, particularly heart failure, raising expectations for its potential value in CAD [5]. Beyond its involvement in inflammation, Gal-3 also regulates tissue fibrosis and angiogenesis [4].

Importantly, Gal-3 is not merely a passive indicator of inflammation but also acts as an active regulator in cardiovascular pathology. It contributes to disease progression through mechanisms including endothelial dysfunction, macrophage infiltration, and fibrotic remodeling, all of which are closely linked to plaque development and progression [6,7]. As a potent pro-inflammatory mediator, Gal-3 promotes macrophage activation, cytokine release, and tissue remodeling [8]. Therefore, circulating Gal-3 concentrations may reflect not only the presence but also the intensity and persistence of inflammation. Although several studies suggest that Gal-3 could serve as a relevant biomarker in cardiovascular diseases [4], direct comparative evidence between ACS and CCS remains scarce. This gap limits its integration into diagnostic and therapeutic algorithms. Accordingly, this study was designed to investigate the biological role of Gal-3 in atherosclerosis, to compare its levels in ACS and CCS patients, and to assess its diagnostic and prognostic significance.

## 2. Materials and Methods

### 2.1. Data Collection

#### 2.1.1. Study Design

This prospective study was conducted at the Department of Cardiology, Mersin University Faculty of Medicine, between August and October 2025. The inclusion criteria were being 18 years of age or older and having undergone coronary angiography (CAG) based on clinical indications. Individuals with active infections, known malignancies, or diagnosed rheumatologic diseases were excluded from the study. Patients who underwent CAG with a preliminary diagnosis of acute coronary syndrome (ACS) or chronic coronary syndrome (CCS) were classified into three groups according to angiographic findings: ACS, CCS, and control. Participants with normal coronary angiography findings were assigned to the control group. It should be emphasized that the control group did not consist of asymptomatic healthy volunteers but rather of individuals referred for coronary angiography due to clinical suspicion of coronary artery disease. Therefore, this group represents a population with similar referral characteristics but without angiographic evidence of obstructive coronary lesions. This design was intentionally chosen to reflect real-world clinical practice; however, it may limit the interpretation of findings in the context of comparisons with truly healthy populations. Acute coronary syndrome was defined according to contemporary guideline-based criteria, including compatible clinical presentation with evidence of myocardial ischemia supported by electrocardiographic changes and/or elevated cardiac biomarkers, and patients were classified as ACS prior to coronary angiography. Subclassification of acute coronary syndrome into ST-elevation myocardial infarction, non-ST-elevation myocardial infarction, and unstable angina was not performed due to sample size considerations, which may have limited the detection of subtype-specific biomarker behavior. Chronic coronary syndrome was defined as stable symptoms and/or objective evidence of ischemia without features of an acute coronary syndrome. The control group consisted of individuals who underwent coronary angiography for clinical indications but had no angiographic evidence of obstructive coronary artery disease.

Sample size estimation was based on the expected between-group difference in Galectin-3 levels derived from a previously published study with a comparable coronary artery disease population. Because the reference study reported central tendency and dispersion measures in a non-parametric format, these values were used to derive an approximate standardized effect size, which corresponded to Cohen’s d = 0.63, representing a moderate effect. Using this estimate, an a priori power analysis was performed for three independent groups within a one-way fixed-effects analysis framework. With a two-sided alpha level of 0.05 and statistical power of 80% (1-β = 0.80), the minimum required total sample size was calculated as 66 participants (22 per group). The power analysis was conducted using G*Power version 3.1.9.7 software. To improve robustness and reduce the risk of type II error, the final study population was expanded to 180 participants, with 60 individuals in each group.

#### 2.1.2. Data Collection

Fasting venous blood samples (5 mL) were collected from all participants in the morning. Serum was separated and stored at −20 °C until analysis. On the day of measurement, Galectin-3 concentrations were determined using the enzyme-linked immunosorbent assay (ELISA) method. Optical density values were interpreted with reference to standard curves. In addition to Galectin-3 measurements, demographic characteristics, clinical risk factors, and laboratory parameters were systematically recorded and analyzed.

### 2.2. Data Analysis

#### 2.2.1. Statistical Analysis

All statistical analyses were conducted according to a predefined analysis plan to ensure methodological rigor and reproducibility. Prior to inferential analysis, data distribution was assessed using both visual (histograms and Q–Q plots) and analytical methods. The Shapiro–Wilk test was used to evaluate the normality of continuous variables due to its high statistical power in moderate sample sizes. Based on these assessments, most continuous variables demonstrated non-normal distribution; therefore, non-parametric statistical methods were preferred.

Continuous variables were expressed as median and interquartile range (IQR), while categorical variables were presented as absolute frequency (n) and percentage (%). Comparisons among the three independent study groups (Control, ACS, and CCS) were performed using the Kruskal–Wallis test for continuous variables. When a statistically significant overall difference was detected, pairwise post hoc comparisons were performed using Bonferroni-adjusted Dunn’s test to control for type I error due to multiple comparisons. Adjustment for multiple testing was primarily applied to pairwise post hoc group comparisons, where the risk of inflated type I error was highest. Accordingly, Bonferroni correction was used following significant omnibus Kruskal–Wallis results. Correlation, ROC, and regression analyses were considered secondary and hypothesis-generating analyses and were therefore interpreted cautiously in the context of effect size, confidence intervals, and overall consistency rather than on nominal *p*-values alone. Categorical variables were compared using the Pearson Chi-square test or Fisher’s exact test when appropriate, based on expected cell frequencies.

Correlation analyses between Galectin-3 levels and continuous clinical and laboratory parameters were performed separately within the ACS and CCS groups using Spearman’s rank correlation coefficient (rho), as this method does not assume normal data distribution and is appropriate for ordinal or non-normally distributed continuous variables. For each correlation coefficient, 95% confidence intervals were estimated using bootstrap resampling with 1000 iterations to provide a more robust assessment of statistical precision. Correlation strength was interpreted according to established thresholds: weak (|r| < 0.30), moderate (0.30–0.49), and strong (≥0.50).

Receiver Operating Characteristic (ROC) curve analysis was performed to evaluate the diagnostic discrimination ability of Galectin-3 for identifying acute coronary syndrome (ACS) and chronic coronary syndrome (CCS) relative to the control group. The area under the ROC curve (AUC) was calculated as a measure of overall diagnostic accuracy. AUC values were interpreted as follows: 0.50–0.59 (no discrimination), 0.60–0.69 (poor), 0.70–0.79 (acceptable), 0.80–0.89 (excellent), and ≥0.90 (outstanding discrimination). The optimal cutoff value was determined using the Youden index (J = sensitivity + specificity − 1), which identifies the threshold that maximizes overall classification performance. Sensitivity, specificity, and their corresponding 95% confidence intervals (CI) were calculated using nonparametric methods. Confidence intervals for ROC curves were estimated using bootstrap resampling with 1000 iterations to improve estimation stability and reduce sampling variability.

To identify independent predictors of ACS and CCS, multivariable logistic regression analysis was performed using the enter method. Clinically relevant variables and those with potential association in univariate analysis were included in the multivariable model. Model results were presented as odds ratios (OR) with corresponding 95% confidence intervals (CI). The absence of multicollinearity among predictor variables was verified using the variance inflation factor (VIF) analysis, with VIF values < 5 considered acceptable. Model goodness-of-fit was assessed using the Hosmer–Lemeshow test. Additional logistic regression assumptions were also evaluated before final model interpretation. The linearity of the logit for continuous predictors was assessed using Box–Tidwell procedures, and no meaningful violation was identified. Model specification was further reviewed for sparse-cell structure and undue influence of individual observations. These steps were undertaken to ensure the stability and interpretability of the regression estimates. To improve interpretability, Galectin-3 was also evaluated after rescaling (per 100 pg/mL increase) in sensitivity analyses. Model calibration, goodness-of-fit, multicollinearity, and linearity assumptions were assessed before final interpretation of the regression models.

All statistical tests were two-tailed, and a *p*-value < 0.05 was considered statistically significant.

#### 2.2.2. Software

All statistical analyses were performed using IBM SPSS Statistics for Windows (Version 29.0; IBM Corp., Armonk, NY, USA). ROC curve analyses were conducted using MedCalc Statistical Software (Version 22; MedCalc Software Ltd., Ostend, Belgium). ROC curve analyses, cutoff value determination using the Youden index, bootstrap confidence interval estimation, and logistic regression modeling were conducted using MedCalc to ensure accurate diagnostic performance evaluation.

## 3. Results

A total of 180 individuals, categorized based on coronary angiography (CAG) findings, were included in this prospective study. Participants were divided into three groups: control (n = 60), acute coronary syndrome (ACS; n = 60), and chronic coronary syndrome (CCS; n = 60). Although the primary aim of the study was to compare Gal-3 levels among the groups, accompanying demographic data, clinical risk factors, and laboratory parameters were also evaluated. Additionally, correlation analyses were conducted to investigate the relationships between Gal-3 levels and other biochemical and clinical parameters. This approach aimed not only to assess Gal-3 as a diagnostic marker, but also to explore whether it may function as a biological mediator associated with clinical parameters.

The baseline characteristics of the study population are summarized in Table 1. Age differed significantly among the three groups, with progressively higher median age values observed from the control group to the CCS group (*p* < 0.001). Renal function, as assessed by GFR, was significantly reduced in both the ACS and CCS groups compared with the control group, with the lowest values observed in the CCS group (*p* < 0.001). This finding is particularly relevant given the established relationship between Galectin-3, renal impairment, and fibrotic processes. Reduced renal clearance and concomitant inflammatory activation may contribute to elevated circulating Galectin-3 levels independent of coronary plaque instability. In addition, residual confounding related to unmeasured factors such as medication use, metabolic syndrome components, obesity, and subclinical inflammatory states cannot be excluded. These variables may have influenced systemic inflammatory burden and biomarker expression, highlighting the need for future studies incorporating more comprehensive phenotypic characterization. Serum sodium levels also showed significant intergroup differences, being lower in the ACS group compared with both the control and CCS groups (*p* < 0.001). Galectin-3 concentrations were markedly higher in both ACS and CCS patients than in controls (*p* < 0.001), whereas BMI, potassium, and LDL cholesterol levels did not differ significantly among groups. HbA1c levels were significantly higher in the CCS group compared with the control group (*p* = 0.010). LVEF differed significantly across groups, with the lowest median value in the ACS group, followed by the CCS group (*p* < 0.001). Regarding comorbidities, the prevalence of diabetes mellitus was higher in the CCS group compared with the control group (*p* = 0.043), while hypertension was more frequent in the CCS group than in the ACS group (*p* = 0.036). Smoking was significantly more common in the ACS group compared with controls (*p* = 0.004). Sex distribution also differed significantly among groups, with a higher proportion of male patients in the CCS group (*p* = 0.006) (Table 1).

The data in Table 2 show that in the ACS group, Galectin-3 levels were positively correlated with age (r = 0.333, *p* = 0.009) and negatively correlated with potassium levels (r = −0.295, *p* = 0.022). In the CCS group, Galectin-3 levels demonstrated a positive correlation with body mass index (r = 0.325, *p* = 0.011) and potassium levels (r = 0.276, *p* = 0.033). The confidence interval analysis demonstrated that only correlations between Galectin-3 and age and potassium in the ACS group, and between Galectin-3 and BMI and potassium in the CCS group, showed intervals not crossing zero, supporting the statistical robustness of these associations (Table 2).

The data in Table 3 show that Galectin-3 demonstrated a moderate diagnostic performance for predicting acute coronary syndrome, with an area under the curve of 0.617. The optimal cut-off value for Galectin-3 was identified as 745.55 pg/mL, yielding a sensitivity of 96.7% and a specificity of 43.3% (Table 3).

To further evaluate the incremental diagnostic contribution of Galectin-3, multivariable ROC analyses were performed using clinical risk factors associated with acute coronary syndrome. A clinical model including age, sex, and smoking demonstrated acceptable discrimination (AUC = 0.701). However, the addition of Galectin-3 to this model resulted in only a minimal change in overall diagnostic performance (AUC = 0.701), indicating limited incremental value beyond established clinical predictors. These findings suggest that although Galectin-3 is associated with the presence of coronary artery disease, its contribution to diagnostic discrimination in a multivariable framework remains modest (Table 4).

The data in Table 5 show that Galectin-3 demonstrated a low diagnostic performance for predicting chronic coronary syndrome, with an area under the curve of 0.541. The optimal cut-off value for Galectin-3 was identified as 530.20 pg/mL, providing a sensitivity of 91.7% and a specificity of 30.0% (Table 4).

**Figure 1:**
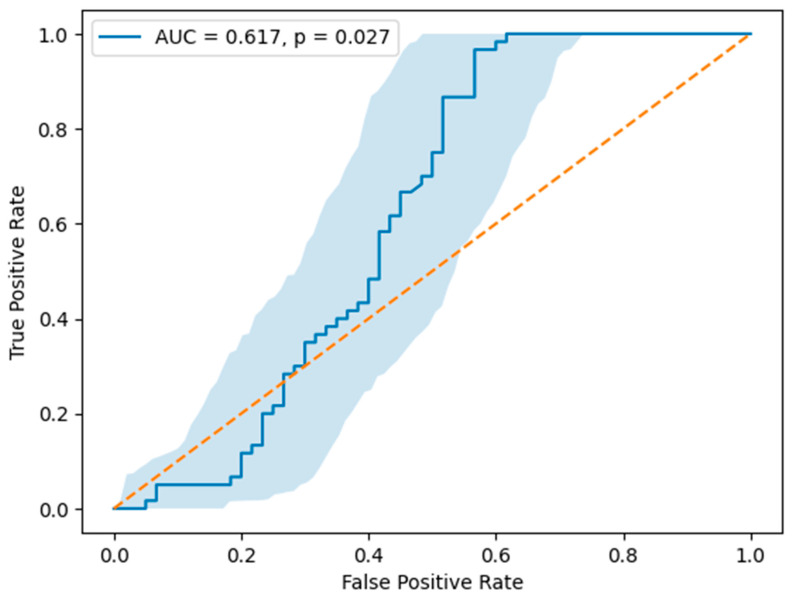
Receiver operating characteristic (ROC) curve of Galectin-3 for the identification of acute coronary syndrome (ACS). The area under the curve (AUC) was 0.617 (*p* = 0.027). The shaded area represents the 95% confidence interval obtained by bootstrap resampling (1000 iterations) (n = 120). The orange diagonal line represents the line of no discrimination.

As shown in Figure 1, receiver operating characteristic analysis demonstrated that Galectin-3 exhibited a modest ability to discriminate patients with acute coronary syndrome from the control group. The area under the curve was 0.617, indicating limited diagnostic accuracy. The ROC analysis yielded a statistically significant result, with a *p*-value of 0.027. The 95% confidence interval of the ROC curve, obtained by bootstrap resampling, showed a broad overlap with the reference line, reflecting considerable variability in discriminatory performance across the range of Galectin-3 values (Figure 1).

**Figure 2:**
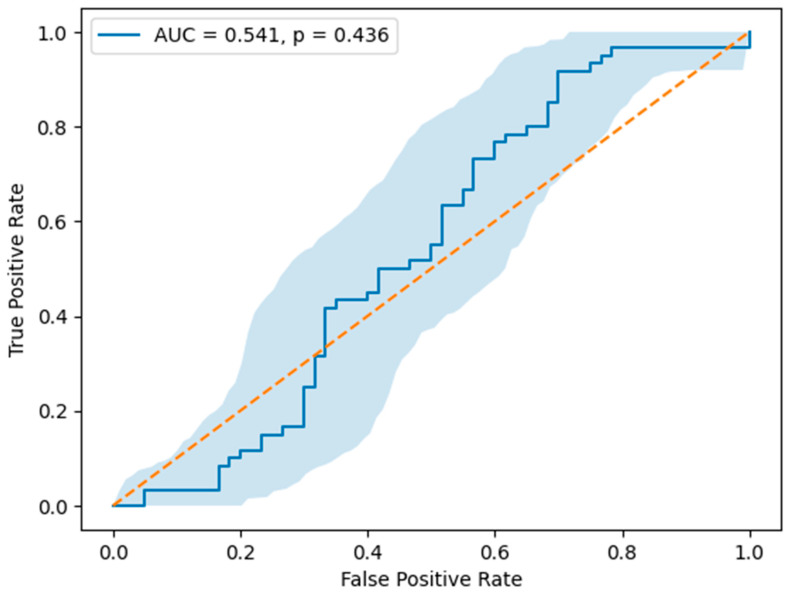
Receiver operating characteristic (ROC) curve of Galectin-3 for the identification of chronic coronary syndrome (CCS). The area under the curve (AUC) was 0.541 (*p* = 0.436). The shaded area represents the 95% confidence interval obtained by bootstrap resampling (1000 iterations) (n = 120). The orange diagonal line represents the line of no discrimination.

As shown in Figure 2, receiver operating characteristic analysis was performed to evaluate the discriminatory ability of Galectin-3 for identifying chronic coronary syndrome. The area under the curve was 0.541, and the analysis did not reach statistical significance (*p* = 0.436). The 95% confidence interval of the ROC curve, generated by bootstrap resampling, demonstrated wide dispersion across the reference line (Figure 2).

The data in Table 6 show that age was independently associated with acute coronary syndrome, with an odds ratio of 1.07 (95% CI: 1.00–1.14, *p* = 0.036). Smoking was also identified as an independent predictor of acute coronary syndrome, with an odds ratio of 2.94 (95% CI: 1.22–7.11, *p* = 0.016) (Table 5).

The data in Table 7 show that age was independently associated with chronic coronary syndrome, with an odds ratio of 1.10 (95% CI: 1.04–1.16, *p* < 0.001). Male sex was also independently associated with chronic coronary syndrome, with an odds ratio of 4.05 (95% CI: 1.66–9.92, *p* = 0.002) (Table 6).

**Figure 3:**
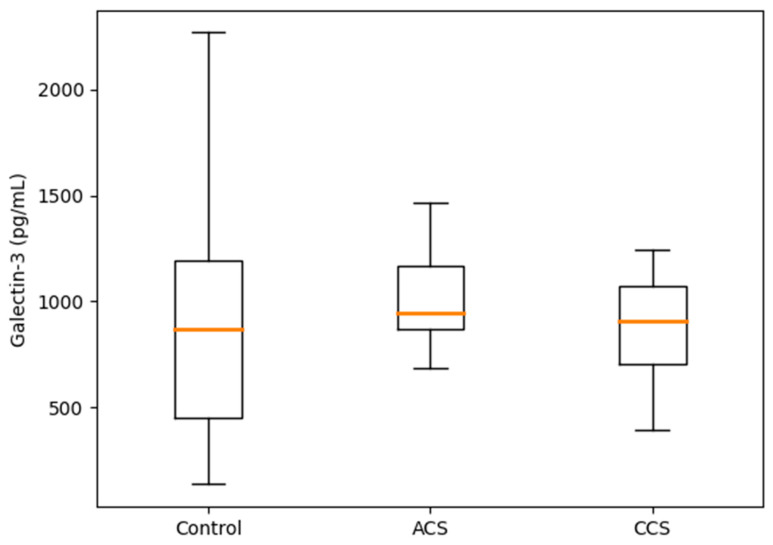
Distribution of Galectin-3 levels across the control, acute coronary syndrome (ACS), and chronic coronary syndrome (CCS) groups. Data are presented as median and interquartile range (IQR). Galectin-3 levels were significantly higher in both the ACS and CCS groups compared with the control group. Overall comparison using the Kruskal–Wallis test demonstrated a statistically significant difference among the three groups (*p* < 0.001) (Control n = 60, ACS n = 60, CCS n = 60; total n = 180). The orange diagonal line represents the line of no discrimination.

As shown in Figure 3, Galectin-3 levels differed significantly among the control, acute coronary syndrome, and chronic coronary syndrome groups. Median Galectin-3 concentrations were higher in both the ACS and CCS groups compared with the control group. Post hoc pairwise comparisons demonstrated that both the ACS and CCS groups had significantly higher Galectin-3 levels than the control group, whereas no statistically significant difference was observed between the ACS and CCS groups. These findings indicate that elevated Galectin-3 levels are associated with the presence of coronary artery disease rather than with the specific clinical stage of the disease. Overall comparison using the Kruskal–Wallis test demonstrated a statistically significant difference in Galectin-3 distribution across the three study groups (*p* < 0.001) (Figure 3).

The data in Table 8 show that the prevalence of hypertension, diabetes mellitus, smoking, and elevated HbA1c levels did not differ significantly between patients with Galectin-3 levels above and below the cut-off value of 745.55 pg/mL (Table 7).

## 4. Discussion

In this prospective angiography-based study, Gal-3 levels were found to be significantly elevated in both acute coronary syndrome (ACS) and chronic coronary syndrome (CCS) patients compared to the control group, while demonstrating limited ability to discriminate between different clinical stages of coronary artery disease. No significant difference in Gal-3 levels was observed between the ACS and CCS groups. Furthermore, correlation analysis revealed a significant positive association between Gal-3 levels and age (r = 0.333, *p* = 0.009) and a significant negative association with potassium levels (r = −0.295, *p* = 0.022) in the ACS group, whereas in the CCS group, Gal-3 levels showed a significant positive correlation with body mass index (r = 0.325, *p* = 0.011) and potassium levels (r = 0.276, *p* = 0.033). These findings indicate that Gal-3 may serve as a biomarker reflecting not only the presence of coronary artery disease but also its association with metabolic and clinical parameters. Another important methodological consideration relates to the definition of the control group. In the present study, control participants were individuals with normal coronary angiography findings rather than completely healthy subjects without cardiovascular symptoms or risk factors. Although this approach improves clinical comparability by minimizing referral bias and reflecting real-world diagnostic pathways, it may also introduce heterogeneity related to underlying subclinical atherosclerosis or systemic inflammatory conditions. Consequently, the interpretation of differences between groups should be made cautiously, particularly when extrapolating results to truly healthy populations.

Coronary artery disease (CAD), which is fundamentally driven by atherosclerotic processes, remains one of the leading causes of cardiovascular morbidity and mortality worldwide, despite advances in modern diagnostic and therapeutic strategies [2]. It is now well established that atherosclerosis is not merely a process of lipid accumulation but a chronic and dynamic pathological condition actively involving inflammatory mechanisms [9]. Recent studies have demonstrated that inflammation plays a central role not only in the formation of atherosclerotic plaques but also in plaque instability, rupture, and subsequent thrombus formation. In this context, inflammation emerges as a key factor not only in coronary artery disease but also in various cardiac pathologies such as heart failure, cardiac fibrosis, and myocardial remodeling [10,11]. Accordingly, biomarkers that reflect inflammation with sensitivity and dynamism hold significant clinical value, particularly in time-sensitive clinical scenarios such as acute coronary syndrome (ACS). Therefore, understanding inflammatory markers and the molecular pathways involved in these processes, as well as targeting them therapeutically, has become increasingly important in the management of cardiovascular diseases.

Galectin-3 is a member of the β-galactoside-binding lectin family, characterized by a single carbohydrate recognition domain and classified as a chimera-type protein. Gal-3 is widely expressed in various cell types, ranging from immune cells to epithelial tissues, and can be found in the cytoplasm, nucleus, cell surface, and biological fluids. Intracellularly, it is involved in the regulation of apoptosis, pre-mRNA splicing, and modulation of survival signaling pathways. Extracellularly, it plays a role in modulating cell–cell and cell–matrix interactions [8].

Additionally, Gal-3 plays a central role in fibrogenesis and inflammatory responses. Its regulatory effects on neutrophil apoptosis, phagocytosis, and T-cell responses indicate its involvement in a wide range of pathological processes—from cardiovascular remodeling to diabetes [3,4]. Through these mechanisms, Gal-3 serves as a key protein in both physiological development and the orchestration of pathological fibro-inflammatory responses [12,13].

The strong correlation between Gal-3 levels and inflammation has drawn attention to its potential role in cardiovascular diseases. This has significantly increased scientific interest in exploring the relationship between Gal-3 and cardiac pathologies. In recent years, Gal-3 has emerged as an increasingly prominent biomarker in the progression of cardiovascular diseases [3,4,12].

A comprehensive review of the literature has reported that Gal-3 levels are considered an independent prognostic marker in both acute coronary syndrome (ACS) and chronic coronary syndrome (CCS) cases [14]. Similarly, in our study, Gal-3 levels were found to be significantly elevated in both ACS and CCS patients compared to healthy individuals, supporting the notion that Gal-3 is a sensitive inflammatory biomarker associated with the presence of disease. In a study conducted by Li et al., Gal-3 levels were shown to be significantly higher in individuals diagnosed with coronary artery disease compared to a healthy control group. Moreover, a positive correlation was identified between Gal-3 levels and the frequency of cardiovascular events [15]. Consistent with the findings of Li et al. [15], our study also demonstrated significantly elevated Gal-3 levels in both ACS and CCS groups compared to the control group. A key novel aspect of the present study is the direct prospective comparison of Galectin-3 levels across distinct clinical stages of coronary artery disease within an angiography-based cohort. In addition to confirming the association between Galectin-3 and the presence of coronary artery disease, our data provide evidence that Galectin-3 lacks both stage-specific discriminatory performance and independent predictive value in multivariable clinical models. These findings extend existing literature by demonstrating that elevated Galectin-3 reflects a shared inflammatory burden rather than clinically actionable diagnostic differentiation between acute and chronic coronary syndromes.

The study conducted by Mayyas and colleagues provides insight into the potential role of Gal-3 in differential diagnosis and risk stratification. In their study, Gal-3 levels were not only significantly higher in individuals with coronary artery disease compared to the control group, but also notably elevated in ACS patients relative to those with stable CCS [16]. This distinction highlights Gal-3’s sensitivity to the acute phase of the disease. However, in our study, no significant difference in Gal-3 levels was observed between ACS and CCS groups. This finding suggests that while Gal-3 is sensitive to the presence of inflammation, it may be limited in distinguishing between different clinical stages of the disease. One possible explanation for this limited discriminatory performance is that Galectin-3 may primarily reflect the overall fibro-inflammatory burden associated with atherosclerosis rather than the acute plaque-rupture and thrombosis biology that distinguishes ACS from CCS. Accordingly, Gal-3 may be more suitable as a marker of disease presence or systemic inflammatory activity, whereas stage discrimination may require integration with other biomarkers and clinical variables in a multimarker framework. Consistent with previous literature, our study also demonstrated that Gal-3 levels were significantly elevated in both ACS and CCS patients compared to healthy controls. Importantly, the present study provides additional clinical insight beyond the existing literature by prospectively evaluating the diagnostic discrimination of Galectin-3 across different clinical stages of coronary artery disease in a real-world cohort undergoing coronary angiography. While previous studies have primarily focused on the association of Galectin-3 with disease presence or long-term prognosis, comparative evidence addressing its stage-specific diagnostic performance remains limited. Our findings demonstrate that although Galectin-3 reflects the inflammatory burden of atherosclerosis, it lacks sufficient discriminatory power to differentiate acute plaque instability from chronic stable disease. This observation has clinical relevance, as it highlights the limitations of relying on a single biomarker in complex inflammatory conditions such as coronary artery disease. Therefore, the novelty of this study lies not only in confirming the association between Galectin-3 and CAD, but also in systematically demonstrating its restricted utility in stage differentiation within a prospective angiography-based population, thereby supporting the need for multimarker strategies in clinical decision-making. This supports the notion that Gal-3 reflects a shared inflammatory response across different clinical phases of atherosclerosis.

Our findings support the role of Gal-3 in the pathogenesis of coronary artery disease (CAD) and confirm its sensitivity to the presence of disease. Furthermore, no statistically significant difference in Gal-3 levels was observed between the ACS and CCS groups in our data. This suggests that although Gal-3 is responsive to the presence of inflammation, it may have limited capacity in differentiating the intensity of inflammatory responses. Alternatively, the heterogeneous inflammatory profiles of patients within the ACS and CCS subgroups may obscure such differences. Therefore, caution is warranted when considering Gal-3 as a biomarker reflective of not only the presence but also the severity of inflammation.

Importantly, receiver operating characteristic analysis demonstrated that Gal-3 exhibited limited diagnostic discrimination for ACS (AUC = 0.617, sensitivity = 96.7%, specificity = 43.3%, *p* = 0.027) and poor diagnostic discrimination for CCS (AUC = 0.541, sensitivity = 91.7%, specificity = 30.0%, *p* = 0.436), indicating that although Gal-3 levels are elevated in disease states, their standalone diagnostic accuracy remains modest. From a clinical standpoint, the ROC profile observed in this study—characterized by very high sensitivity but poor specificity—suggests limited real-world applicability of Galectin-3 as a standalone diagnostic biomarker. Such performance may lead to substantial false-positive classification, potentially resulting in unnecessary invasive testing, increased healthcare burden, and patient anxiety. Moreover, the absence of meaningful incremental diagnostic improvement in multimarker models further supports the notion that Galectin-3 cannot substitute established biomarkers such as cardiac troponins in acute coronary syndromes or inflammatory indicators such as C-reactive protein. Instead, Galectin-3 may be more appropriately interpreted as a marker of systemic inflammatory activity rather than a clinically actionable discriminator of disease stage. Importantly, multimarker diagnostic modeling demonstrated that the integration of Galectin-3 into a clinical risk model including age, sex, and smoking did not result in a meaningful improvement in discriminatory performance. This observation highlights a critical concept in contemporary cardiovascular biomarker research: while inflammatory markers may reflect underlying disease biology, their incremental diagnostic utility beyond established clinical predictors may be limited. Therefore, Galectin-3 appears to function primarily as a marker of systemic inflammatory burden rather than as a stage-specific diagnostic discriminator. These findings support the growing emphasis on integrated risk assessment strategies combining clinical, biochemical, and imaging parameters for more accurate characterization of coronary artery disease. Furthermore, multivariable logistic regression analysis showed that Gal-3 was not an independent predictor of ACS (OR = 0.9999, *p* = 0.597) or CCS (OR = 1.0000, *p* = 0.860), whereas age and smoking were independently associated with ACS, and age and male sex were independently associated with CCS.

In recent years, Gal-3 has gained attention as a significant biomarker reflecting increased inflammatory burden and metabolic disturbances in diabetes and diabetes-related complications [17]. Studies have shown that Gal-3 is involved in the pathogenesis of both Type 1 and Type 2 diabetes through various mechanisms [18]. Macrophage-derived Gal-3 has been reported to infiltrate pancreatic islets, intensify inflammatory responses, accelerate β-cell death, and impair insulin secretion, thereby negatively affecting glycemic control [19]. Additionally, Gal-3 has been shown to interact directly with the insulin receptor, inhibiting insulin signaling in peripheral tissues and contributing to insulin resistance through this mechanism [17]. At the pancreatic β-cell level, Gal-3 has been found to interfere with intracellular calcium flux by interacting with CACNG1, a calcium channel subunit on the cell membrane, thereby significantly reducing glucose-stimulated insulin secretion [19]. This impairment leads to a marked decline in β-cell function, while genetic or pharmacological inhibition of Gal-3 has been shown to significantly improve insulin secretion and glucose tolerance [20]. In a clinical observational study involving individuals with type 2 diabetes, Gal-3 levels were positively correlated with microvascular complications of diabetes (retinopathy, neuropathy, nephropathy) and were significantly elevated in conjunction with metabolic disturbances such as elevated HbA1c and dyslipidemia [21].

In contrast to some previous studies reporting strong metabolic correlations, our findings demonstrated that Gal-3 was associated with selected clinical and biochemical parameters rather than glycemic markers, suggesting that its clinical significance may reflect a broader inflammatory and systemic response rather than isolated metabolic dysfunction.

In recent years, the growing scientific interest in cardioinflammatory processes has highlighted that Gal-3 is not only a biomarker but also an active molecular mediator directly involved in disease pathogenesis. In light of current findings, beyond its diagnostic sensitivity, Gal-3’s capacity to predict disease prognosis and its potential as a therapeutic target are gaining increasing significance. However, given its limited diagnostic accuracy and lack of independent predictive value in multivariable analysis, its integration into routine clinical decision-making should be approached cautiously. Large-scale, long-term prospective studies involving heterogeneous patient populations are needed to better define its clinical utility.

### Limitations of the Study

This study has several methodological and clinical limitations. First, although the sample size was increased to 180 participants, the division of participants into three distinct groups may still limit the statistical power for detecting subtle subgroup-specific associations. Additionally, the study was conducted in a single center with a patient population characterized by specific geographic and sociodemographic features. This limits the generalizability of the findings to patients in other settings or broader populations. Furthermore, the control group consisted of patients undergoing coronary angiography for clinical indications rather than asymptomatic healthy individuals. Although the absence of angiographically significant coronary stenosis defined the control status, these individuals may still have had cardiovascular risk factors, microvascular disease, or early-stage atherosclerosis not detectable by conventional angiography. This methodological aspect may have attenuated the observed differences between groups and should be considered when interpreting the diagnostic performance of Galectin-3.

Second, due to the cross-sectional design of the study, only concurrent associations with Gal-3 levels were assessed. As a result, the long-term impact of Gal-3 on disease prognosis—such as mortality, major adverse cardiovascular events (MACE), or rehospitalization—was not evaluated. Consequently, it remains uncertain whether Galectin-3 has prognostic value despite its limited diagnostic discrimination in the present cross-sectional analysis. This represents a limitation in establishing the prognostic value of Gal-3.

Third, other inflammatory biomarkers known to influence Gal-3 levels (e.g., CRP, IL-6, TNF-α) were not simultaneously assessed. This limited the ability to determine whether Gal-3 provides independent clinical value beyond established inflammatory markers. Similarly, comorbid conditions that may contribute to systemic inflammation—such as rheumatologic diseases, infections, or chronic kidney disease—were not comprehensively excluded; only clinically overt cases were omitted. This may have limited control over external factors influencing Gal-3 levels.

Fourth, although multivariable regression analysis was performed, Gal-3 was not identified as an independent predictor of ACS or CCS, and residual confounding factors cannot be completely excluded. The absence of longitudinal follow-up data also limits the evaluation of its prognostic or predictive utility.

Finally, the cross-sectional nature of the study does not allow for causal inference. To determine whether Gal-3 levels are associated not only with the presence but also with the stage, duration, or activity of the disease, prospective studies that monitor temporal changes are required.

For all these reasons, the current findings should be interpreted with caution. The integration of Gal-3 into clinical practice requires further advanced studies that are multicenter, large-scale, prospective, and supported by long-term follow-up data.

## 5. Conclusions

In this prospective observational study, Galectin-3 levels were significantly elevated in patients with coronary artery disease compared with angiographically normal individuals. However, the biomarker demonstrated poor discriminatory performance between acute and chronic clinical presentations and did not retain independent predictive value after adjustment for conventional clinical variables. These findings suggest that although Galectin-3 reflects systemic inflammatory and fibrotic activity, it lacks sufficient clinical utility as a standalone diagnostic biomarker in this population. Future research should focus on longitudinal prognostic assessment and multimarker strategies integrating clinical, biochemical, and imaging parameters.

## Figures and Tables

**Table 1:** Baseline Characteristics of the Study Groups (n = 180).

Variable	Control (n = 60)	ACS (n = 60)	CCS (n = 60)	*p*-Value
Age (years)	56.00 [51.00–59.00]	59.00 [55.00–64.00]	61.00 [56.00–67.00]	**<0.001**
BMI (kg/m^2^)	28.54 [26.32–30.10]	26.66 [24.68–28.55]	27.25 [26.05–30.32]	0.051
GFR (mL/min/1.73 m^2^)	103.00 [94.50–111.50]	96.50 [90.00–101.25] ^a^	90.50 [73.00–99.00] ^ab^	**<0.001**
Sodium (mmol/L)	140.00 [138.00–141.00]	136.00 [134.75–138.00] ^a^	139.00 [137.00–142.00] ^b^	**<0.001**
Potassium (mmol/L)	4.30 [4.00–4.50]	4.00 [3.88–4.32]	4.25 [3.80–4.50]	0.103
LDL cholesterol (mg/dL)	108.00 [84.00–126.25]	118.50 [93.75–134.00]	102.00 [77.75–146.25]	0.523
Galectin-3 (pg/mL)	871.33 [453.23–1195.29]	942.87 [869.50–1167.65] ^a^	908.28 [703.76–1070.80] ^a^	**<0.001**
HbA1c (%)	5.90 [5.57–6.12]	5.90 [5.55–6.50]	5.80 [5.50–7.20] ^a^	**0.010**
LVEF (%)	60.00 [60.00–60.00]	50.00 [45.00–55.00] ^a^	55.00 [52.25–55.25] ^b^	**<0.001**
Diabetes mellitus, n (%)	13 (21.7)	20 (33.3)	21 (35.0) ^a^	**0.043**
Hypertension, n (%)	25 (41.7)	32 (53.3)	26 (43.3) ^b^	**0.036**
Hyperlipidemia, n (%)	13 (21.7)	16 (26.7)	28 (46.7)	0.081
Smoking, n (%)	24 (40.0)	42 (70.0) ^a^	30 (50.0)	**0.004**
Female sex, n (%)	33 (55.0)	32 (53.3)	29 (48.3)	**0.006**
Male sex, n (%)	27 (45.0)	28 (46.7)	31 (51.7) ^a^	—

Statistical analyses were performed using the Kruskal–Wallis test with Bonferroni-adjusted post hoc comparisons for non-normally distributed continuous variables and the Chi-square test for categorical variables. Continuous variables are presented as median [interquartile range], and categorical variables as number (%). A *p*-value < 0.05 was considered statistically significant. BMI = Body Mass Index, GFR = Glomerular Filtration Rate, LDL = Low-Density Lipoprotein, LVEF = Left Ventricular Ejection Fraction. ^a^: Significantly different from the control group (Bonferroni-adjusted *p* < 0.05). ^b^: Significantly different from the ACS group (Bonferroni-adjusted *p* < 0.05). Statistically significant *p*-values are marked in bold.

**Table 2:** Correlation Between Galectin-3 Levels and Clinical Parameters (ACS n = 60, CCS n = 60).

Variable	ACS (r)	95% CI	*p*-Value	CCS (r)	95% CI	*p*-Value
Age	0.333	0.08 to 0.55	0.009	0.240	−0.02 to 0.46	0.065
BMI	0.217	−0.05 to 0.45	0.095	0.325	0.06 to 0.54	**0.011**
GFR	−0.036	−0.28 to 0.22	0.782	−0.237	−0.46 to 0.02	0.068
Sodium	0.066	−0.19 to 0.31	0.614	0.101	−0.16 to 0.35	0.441
Potassium	−0.295	−0.52 to −0.05	**0.022**	0.276	0.02 to 0.49	**0.033**
LDL cholesterol	−0.104	−0.34 to 0.16	0.430	−0.114	−0.35 to 0.15	0.386
HbA1c	0.172	−0.09 to 0.41	0.190	−0.133	−0.37 to 0.12	0.312
LVEF	0.151	−0.10 to 0.39	0.251	0.239	−0.02 to 0.46	0.066

Statistical tests applied include Spearman’s rank correlation analysis to evaluate the relationship between Galectin-3 levels and continuous clinical variables in the ACS and CCS groups. Correlation coefficients (r) are presented together with their corresponding 95% confidence intervals estimated using bootstrap resampling with 1000 iterations. A *p*-value < 0.05 was considered statistically significant. BMI = Body Mass Index, GFR = Glomerular Filtration Rate, LDL = Low-Density Lipoprotein, HbA1c = Hemoglobin A1c, LVEF = Left Ventricular Ejection Fraction. Statistically significant *p*-values are marked in bold.

**Table 3:** ROC Curve Analysis of Galectin-3 for Predicting Acute Coronary Syndrome (ACS) (n = 120 (ACS n = 60, Control n = 60)).

Parameter	AUC	Cut-Off (pg/mL)	Sensitivity (%)	Specificity (%)
Galectin-3	0.617	745.55	96.7	43.3

Statistical tests applied include receiver operating characteristic (ROC) curve analysis to evaluate the diagnostic performance of Galectin-3 for predicting acute coronary syndrome. Optimal cut-off values were determined using the Youden index (J = sensitivity + specificity − 1). The *p*-value indicates the level of statistical significance, with values less than 0.05 considered statistically significant. AUC = Area Under the Curve.

**Table 4:** Incremental Diagnostic Performance of Galectin-3 in Multivariable Models for Predicting Acute Coronary Syndrome.

Model	Variables Included	AUC	Sensitivity (%)	Specificity (%)
Model 1	Galectin-3	0.617	96.7	43.3
Model 2	Age + Sex + Smoking	0.701	68.3	65.0
Model 3	Age + Sex + Smoking + Galectin-3	0.701	70.0	63.3

Receiver operating characteristic (ROC) curve analysis was performed to evaluate the incremental diagnostic value of Galectin-3 beyond conventional clinical risk factors. Multivariable probability scores derived from logistic regression models were used to construct ROC curves. The area under the curve (AUC) was used to assess overall discrimination performance. Sensitivity and specificity values were calculated at optimal cutoff thresholds determined by the Youden index. Age was analyzed as a continuous variable, while sex and smoking status were treated as categorical variables.

**Table 5:** ROC Curve Analysis of Galectin-3 for Predicting Chronic Coronary Syndrome (CCS) (n = 120 (CCS n = 60, Control n = 60)).

Parameter	AUC	Cut-Off (pg/mL)	Sensitivity (%)	Specificity (%)
Galectin-3	0.541	530.20	91.7	30.0

Statistical tests applied include receiver operating characteristic (ROC) curve analysis to evaluate the diagnostic performance of Galectin-3 for predicting chronic coronary syndrome. Optimal cut-off values were determined using the Youden index (J = sensitivity + specificity − 1). The *p*-value indicates the level of statistical significance, with values less than 0.05 considered statistically significant. AUC = Area Under the Curve.

**Table 6:** Multivariable Logistic Regression Analysis for Predicting Acute Coronary Syndrome (n = 120).

Variable	OR	95% CI	*p*-Value
Galectin-3 (pg/mL)	0.9999	0.9995–1.0003	0.597
Age (years)	1.07	1.00–1.14	**0.036**
Male sex	1.00	0.44–2.23	0.994
Diabetes mellitus	1.08	0.31–3.72	0.899
Hypertension	0.43	0.16–1.11	0.081
Smoking	2.94	1.22–7.11	**0.016**
HbA1c (%)	1.32	0.81–2.17	0.265

Statistical tests applied include multivariable logistic regression analysis to identify independent predictors of acute coronary syndrome in comparison with the control group. The regression model included Galectin-3, age, sex, diabetes mellitus, hypertension, smoking status, and HbA1c. In the table, statistically significant values are marked in bold. The *p*-value indicates the level of statistical significance, with values less than 0.05 considered statistically significant. OR = Odds Ratio, CI = Confidence Interval.

**Table 7:** Multivariable Logistic Regression Analysis for Predicting Chronic Coronary Syndrome (n = 120).

Variable	OR	95% CI	*p*-Value
Galectin-3 (pg/mL)	1.0000	0.9997–1.0004	0.860
Age (years)	1.10	1.04–1.16	**<0.001**
Male sex	4.05	1.66–9.92	**0.002**
Diabetes mellitus	1.13	0.29–4.43	0.856
Hypertension	1.09	0.41–2.89	0.863
Smoking	1.98	0.78–5.02	0.153
HbA1c (%)	1.52	0.83–2.78	0.175

Statistical tests applied include multivariable logistic regression analysis to identify independent predictors of chronic coronary syndrome in comparison with the control group. The regression model included Galectin-3, age, sex, diabetes mellitus, hypertension, smoking status, and HbA1c. In the table, statistically significant values are marked in bold. The *p*-value indicates the level of statistical significance, with values less than 0.05 considered statistically significant. OR = Odds Ratio, CI = Confidence Interval, statistically significant *p*-values are indicated in bold.

**Table 8:** Distribution of Cardiometabolic Risk Factors According to Galectin-3 Cut-off (Galectin-3 ≥ 745.55 pg/mL n = 136, Galectin-3 < 745.55 pg/mL n = 44 (total n = 180)). **Galectin-3 cut-off: 745.55 pg/mL**. (ROC–Youden index, ACS vs. Control).

Variable	Galectin-3 ≥ 745.55 pg/mL	Galectin-3 < 745.55 pg/mL	*p*-Value
Hypertension, n (%)	80 (58.8)	25 (56.8)	0.953
Diabetes mellitus, n (%)	43 (31.6)	14 (31.8)	1.000
Smoking, n (%)	74 (54.4)	22 (50.0)	0.737
HbA1c ≥ 6.5%, n (%)	38 (27.9)	9 (20.5)	0.432

Statistical tests applied include receiver operating characteristic (ROC) curve analysis with Youden index to determine the optimal Galectin-3 cut-off value and the Chi-Square test for comparisons of categorical variables. The *p*-value indicates the level of statistical significance, with values less than 0.05 considered statistically significant. HbA1c = Hemoglobin A1c.

## Data Availability

The data that support the findings of this study are available from the corresponding author upon reasonable request. The data are not publicly available due to privacy and ethical restrictions related to patient information.

## Abbreviations

The following abbreviations are used in this manuscript:

ACS	Acute Coronary Syndrome
CCS	Chronic Coronary Syndrome
CAD	Coronary Artery Disease
Gal-3	Galectin-3
ROC	Receiver Operating Characteristic
AUC	Area Under the Curve
ELISA	Enzyme-Linked Immunosorbent Assay
BMI	Body Mass Index
GFR	Glomerular Filtration Rate
LDL	Low-Density Lipoprotein
HbA1c	Hemoglobin A1c
LVEF	Left Ventricular Ejection Fraction
OR	Odds Ratio
CI	Confidence Interval
